# Supplementary material for: Pharmacy and neighborhood-level variation in cash price of diabetes medications in the United States
Source: PLoS One. 2023 Dec 7;18(12):e0294164. doi: 10.1371/journal.pone.0294164 (PMC10703254; doi:10.1371/journal.pone.0294164)
Supplement: Attachment — Submitted filename: Reviewers PLOS ONE diabetes.docx [file pone.0294164.s001.docx]

Reviewers’ comments PLOS ONE Pharmacy and neighborhood-level variation in price of diabetes medications in the US

Comments:

1. Authors gave a good rationale for the study- differences in prices could affect those without insurance, other cash paying patients and possibly those with deductibles. I have some comments for clarification and/or consideration.
2. Can you give a reference for the definition of U&C, I saw a reference by Joey Mattingly that the law 42C.F.R &447.512(b) requires pharmacies to report the U&C when submitting pharmacy claims for both Rx and OTC but not cash paying sales?
3. The authors divided the diabetic medications into 3 groups: insulins, non-insulin generics, and brand name non-insulins. What about brand name drugs that are not GLP1s, SGLTs and DPP4s, such as Glucophage? Or brand name insulins? Were they eliminated from the study?
4. I think cash PPU is a poor measure to compare these drugs since collectively, they are given at different doses and frequencies (some are once a week, twice a day etc) why don’t you ‘cost per day’? Or by class, such as generic biguanides, brand name biguanides, GLP1s etc? The variability will be significantly different.
5. Can you define chain pharmacies? Do you mean stand-alone pharmacies like CVS or does it include supermarket chains like Walmart? These 2 chain-types have different pricing strategies and should be treated differently.
6. Can you add the rationale for choosing cutoff for extreme outliers at 10X the median?
7. Do you have a reference for how you treated variability across neighborhoods on page 5?
8. Typo: Tablet instead of table (page 5 last line)
9. Please use generic names instead of brand names like Lantus and Jardiance.
10. Because of how broadly you classified the drugs into 3 groups- it weakens the relevance of the findings. It will be better to treat the drugs by class and that will be meaningful for prescribers.
