# Supplementary material for: Pharmacy and neighborhood-level variation in cash price of diabetes medications in the United States
Source: PLoS One. 2023 Dec 7;18(12):e0294164. doi: 10.1371/journal.pone.0294164 (PMC10703254; doi:10.1371/journal.pone.0294164)
Supplement: Attachment — Submitted filename: Response to Reviewers 7-13.docx [file pone.0294164.s002.docx]

**Journal Requirements:**

**When submitting your revision, we need you to address these additional requirements.**

**1. Please ensure that your manuscript meets PLOS ONE's style requirements, including those for file naming. The PLOS ONE style templates can be found at**

[**https://journals.plos.org/plosone/s/file?id=wjVg/PLOSOne_formatting_sample_main_body.pdf**](https://journals.plos.org/plosone/s/file?id=wjVg/PLOSOne_formatting_sample_main_body.pdf) **and** 
[**https://journals.plos.org/plosone/s/file?id=ba62/PLOSOne_formatting_sample_title_authors_affiliations.pdf**](https://journals.plos.org/plosone/s/file?id=ba62/PLOSOne_formatting_sample_title_authors_affiliations.pdf)

**-** Reviewed the requirements, thank you

**2. Thank you for stating the following in the Acknowledgments Section of your manuscript:**

**"..MV has received research grant support or served on advisory boards for American Regent, Amgen, AstraZeneca, Bayer AG, Baxter Healthcare, Boehringer Ingelheim, Cytokinetics, Lexicon Pharmaceuticals, Novartis, Pharmacosmos, Relypsa, Roche Diagnostics, and Sanofi, speaker engagements with Novartis and Roche Diagnostics, and participates on clinical trial committees for studies sponsored by Galmed, Novartis, Occlutech, and Impulse Dynamics.."**

**We note that you have provided funding information that is not currently declared in your Funding Statement. However, funding information should not appear in the Acknowledgments section or other areas of your manuscript. We will only publish funding information present in the Funding Statement section of the online submission form.**

**Please remove any funding-related text from the manuscript and let us know how you would like to update your Funding Statement. Currently, your Funding Statement reads as follows:**

**"The author(s) received no specific funding for this work."**

**Please include your amended statements within your cover letter; we will change the online submission form on your behalf.**

- We have added amended statements in the cover letter

**3. Thank you for stating the following in the Competing Interests:**

**"HW is a member of the GoodRx medical advisory board. DL and JvM are employees of GoodRx. MV has received research grant support or served on advisory boards for American Regent, Amgen, AstraZeneca, Bayer AG, Baxter Healthcare, Boehringer Ingelheim, Cytokinetics, Lexicon Pharmaceuticals, Novartis, Pharmacosmos, Relypsa, Roche Diagnostics, and Sanofi, speaker engagements with Novartis and Roche Diagnostics, and participates on clinical trial committees for studies sponsored by Galmed, Novartis, Occlutech, and Impulse Dynamics."**

**We note that one or more of the authors have an affiliation to the commercial funders of this research study : GoodRx, American Regent, Amgen, AstraZeneca, Bayer AG, Baxter Healthcare, Boehringer Ingelheim, Cytokinetics, Lexicon Pharmaceuticals, Novartis, Pharmacosmos, Relypsa, Roche Diagnostics, Sanofi, Roche Diagnostics, Galmed, Occlutech, and Impulse Dynamics.**

**(1) Please provide an amended Funding Statement declaring this commercial affiliation, as well as a statement regarding the Role of Funders in your study. If the funding organization did not play a role in the study design, data collection and analysis, decision to publish, or preparation of the manuscript and only provided financial support in the form of authors' salaries and/or research materials, please review your statements relating to the author contributions, and ensure you have specifically and accurately indicated the role(s) that these authors had in your study. You can update author roles in the Author Contributions section of the online submission form.**

**Please also include the following statement within your amended Funding Statement. 
“The funder provided support in the form of salaries for authors [insert relevant initials], but did not have any additional role in the study design, data collection and analysis, decision to publish, or preparation of the manuscript. The specific roles of these authors are articulated in the ‘author contributions’ section.”**

**If your commercial affiliation did play a role in your study, please state and explain this role within your updated Funding Statement.**

- We have updated this as indicated in the cover letter.

**(2) Please also provide an updated Competing Interests Statement declaring this commercial affiliation along with any other relevant declarations relating to employment, consultancy, patents, products in development, or marketed products, etc.**

**Within your Competing Interests Statement, please confirm that this commercial affiliation does not alter your adherence to all PLOS ONE policies on sharing data and materials by including the following statement: ""This does not alter our adherence to  PLOS ONE policies on sharing data and materials.” (as detailed online in our guide for authors** [**http://journals.plos.org/plosone/s/competing-interests**](http://journals.plos.org/plosone/s/competing-interests)**). If this adherence statement is not accurate and  there are restrictions on sharing of data and/or materials, please state these. Please note that we cannot proceed with consideration of your article until this information has been declared.**

**Please include both an updated Funding Statement and Competing Interests Statement in your cover letter. We will change the online submission form on your behalf.**

- We have added these to the updated cover letter

**4. We note that you have indicated that data from this study are available upon request. PLOS only allows data to be available upon request if there are legal or ethical restrictions on sharing data publicly. For more information on unacceptable data access restrictions, please see** [**http://journals.plos.org/plosone/s/data-availability#loc-unacceptable-data-access-restrictions**](http://journals.plos.org/plosone/s/data-availability#loc-unacceptable-data-access-restrictions)**.**

**In your revised cover letter, please address the following prompts:**

**a) If there are ethical or legal restrictions on sharing a de-identified data set, please explain them in detail (e.g., data contain potentially sensitive information, data are owned by a third-party organization, etc.) and who has imposed them (e.g., an ethics committee). Please also provide contact information for a data access committee, ethics committee, or other institutional body to which data requests may be sent.**

**b) If there are no restrictions, please upload the minimal anonymized data set necessary to replicate your study findings as either Supporting Information files or to a stable, public repository and provide us with the relevant URLs, DOIs, or accession numbers. For a list of acceptable repositories, please see** [**http://journals.plos.org/plosone/s/data-availability#loc-recommended-repositories**](http://journals.plos.org/plosone/s/data-availability#loc-recommended-repositories)**.**

**We will update your Data Availability statement on your behalf to reflect the information you provide.

[Note: HTML markup is below. Please do not edit.]**

- We have updated this in the cover letter

**Reviewers' comments:

Reviewer's Responses to Questions**

**Comments to the Author

1. Is the manuscript technically sound, and do the data support the conclusions?

The manuscript must describe a technically sound piece of scientific research with data that supports the conclusions. Experiments must have been conducted rigorously, with appropriate controls, replication, and sample sizes. The conclusions must be drawn appropriately based on the data presented.**

**Reviewer #1: Partly**

**Reviewer #2: Partly**

**2. Has the statistical analysis been performed appropriately and rigorously?**

**Reviewer #1: No**

**Reviewer #2: Yes**

**3. Have the authors made all data underlying the findings in their manuscript fully available?

The** [**PLOS Data policy**](http://secure-web.cisco.com/1EHHK0ovnm_wCVOKiEIJUHJBibq6zkHhJZ2L-7xdSuurzZ5IgUVtOwiheX13k5mK-QyYCwVnZTJLP7YojuGsZVPxCGYEBYGv3z0JgyU63km5Ia4VQUhk0ONBfHWI5hSdGmTpMb1kI6QSM0QjCyYymwvRWF3WOL-FaY0yobJ810cdoo-VUnROs4yCctwR5d0oLal_lpNNOhbU2lJ80wFfwdxhmMOaW_zZQD2hFlJ6Av1wfxENdOxb-d1vLa3Hpy1cHrpZweDb9daKXaBO3mUseVvN3mZdN9TUK12zjsONRfkFNkcgMPUYXHn53k4P3H_ad/http%3A%2F%2Fwww.plosone.org%2Fstatic%2Fpolicies.action%23sharing) **requires authors to make all data underlying the findings described in their manuscript fully available without restriction, with rare exception (please refer to the Data Availability Statement in the manuscript PDF file). The data should be provided as part of the manuscript or its supporting information, or deposited to a public repository. For example, in addition to summary statistics, the data points behind means, medians and variance measures should be available. If there are restrictions on publicly sharing data—e.g. participant privacy or use of data from a third party—those must be specified.**

**Reviewer #1: No**

**Reviewer #2: No**

**4. Is the manuscript presented in an intelligible fashion and written in standard English?

PLOS ONE does not copyedit accepted manuscripts, so the language in submitted articles must be clear, correct, and unambiguous. Any typographical or grammatical errors should be corrected at revision, so please note any specific errors here.**

**Reviewer #1: Yes**

**Reviewer #2: Yes**

**5. Review Comments to the Author

Please use the space provided to explain your answers to the questions above. You may also include additional comments for the author, including concerns about dual publication, research ethics, or publication ethics. (Please upload your review as an attachment if it exceeds 20,000 characters)**

**Reviewer #1: This study aimed to examine pharmacy and neighborhood factors associated with cost variation for diabetes drugs in the US using all-payer US pharmacy data from 45,874 chain and independent pharmacies reflecting 7,073,909 deidentified claims. The authors reported that cash prices for diabetes medications in the US can vary considerably and that the greatest degree of price variation occurs in non-insulin generic therapies. Overall, it’s topically interesting and timely as the authors properly stated that “high medication costs place a significant burden on the health system and represent an important barrier to broad and equitable prescription drug access.” The reviewer has four main comments regarding conceptualization, methodology, interpretation of the results, and limitations.**

**1. Conceptualization: the authors stated that “The U&C price is the price charged to a customer in the absence of insurance or negotiated discount. Although most customers have prescription medication coverage and do not pay the U&C price, the U&C price is the most visible price at the retail level and impacts those lacking insurance. “(Methods, Page 4) Conceptually, the U&C price is not the price a consumer pays for the medication if the consumer has health insurance, and most US consumers have certain insurance coverage such as Medicare, Medicaid, and private insurance. Hence, the “price” the authors referred to was not the actual price most consumers were paying, and its implication to practice and policy is unclear. A well-thought-out introduction of the U&C price in the light of transactions among the wholesaler, pharmacy chains, consumers with or without insurance is needed to help the - readers to better understand the relevance and significance of the study.**

- We have now significantly expanded our discussion of pricing, adding “These data reflect transactions that were submitted to health insurers and pharmacy benefit managers, with pharmacies reporting the usual and customary (U&C) price for the medication with each transaction. The U&C price is the lowest price a customer might have to pay in the absence of insurance and is also referred to as “cash price”. This price is inclusive of all discounts. Pharmacies are required to report the U&C price which is also what they charge Medicare. Although most customers have prescription medication coverage and do not pay the U&C price, the U&C price is the most visible price at the retail level and impacts those lacking insurance. Pharmacies can respond to their customer needs by adjusting this price. Other prices such as the list price and the average wholesale price are constant across the country, while negotiated prices between PBMs and pharmacies are not publicly available and do not necessarily reflect what the customer ends up paying.”

**2. Methodology: the authors stated that “A generalized linear model with gamma distribution and log link function was constructed for each drug group and the models were stratified by pharmacy type (independent or chain) (Methods, Page 5). Were family (gamma) and link (log) tests performed to determine the appropriateness of the gamma distribution and log link function? How did the distribution of cash price-per-unit (PPU) look like? Some tables/figures would be informative to show the distribution of the outcome measure the authors had in the analysis by drugs.**

- Thank you for your important comment. We have now added further material adding context to our methods, adding “The cash price PPU data is a long right-skewed distribution. Log-link represented a natural choice for our cash price data, where residuals increase with x, and we expected errors to be proportional with the conditional mean. Our approach was derived using the methods outlined by Manning and Mullahy in their paper on estimating log models.(1) From the log link, we performed the modified park test, a modification of the heteroscedasticity test that is modified to help determine the family of distributions to use for a log link function. The test indicated that the Gamma family should be used.”

**3. Interpretation of the results: the authors stated that “We then identified pharmacy and neighborhood factors associated with drug price variation” using the generalized linear model (Methods, Page 5). The authors also stated that “Neighborhood variables sourced from the 2019 American Community Survey included mean household income (normalized), insurance coverage (Medicare, Medicaid, other insurance, no insurance coverage), race/ethnicity (Hispanic/Latino), and mean-to-median household income ratio.” In Results, the analysis was then interpreted as “Average proportion covered by commercial insurance was 53%, followed by Medicaid (18%), Medicare (13%), other insurance (8%) and no insurance (8%).” (Results, Page 6). Was this insurance coverage derived from the ACS or the pharmacy data? Please clarify.**

- Thank you for your note. The insurance coverage was derived from the ACS data. Results, Page 6, explains the average neighborhood characteristics across all include ZCTA3s in the study. We clarified our result in the text by adding that it is the population insurance proportions.

**Please also clarify the unit of analysis, for example, how the neighborhood was defined, how many neighborhoods were covered in this data, and the reference groups in the results, for example, when the authors stated that “Chain pharmacies were likely to have higher prices in neighborhoods with greater proportions of high-income residents (+7.9%), urban housing units (+8.4%), and Medicaid beneficiaries (+17.3%), and had lower prices in neighborhoods with greater proportion of people without insurance (-11.2%),” (Results, Page 7), please clarify the reference groups for each of these comparisons, and where those coefficients came from, their associated p-values, for example, was this +7.9% associated with each unit increase in the household income by ACS, if so, what is the unit increase of household income?**

- We have now significantly clarified these results and added details both regards to the reference groups (1^st^ vs 3rd quartiles) and p values. Specifically we have added “Several neighborhood variables were significantly associated with change in cash prices per PPU when comparing the 1^st^ quartile and the 3^rd^ quartile for that covariate within each pharmacy stratum. **(Table)** Insulin cash prices were lower in independent pharmacies with greater proportion of White residents (-6.7%) and urban housing units (-7.6%) (both p<0.001). Brand name prices were lower in areas with more urban housing units (-7.0%, p<0.001). Chain pharmacies had both lower mean prices and lesser variation compared to independent pharmacies.

The greatest degree of price variation between the 1^st^ and 3^rd^ quartile of the neighborhood covariates analyzed was noted for generic therapies. Chain pharmacies were likely to have higher prices in neighborhoods with greater proportions of high-income residents (+7.9%), urban housing units (+8.4%), and Medicaid beneficiaries (+17.3%), and had lower prices in neighborhoods with greater proportion of people without insurance (-11.2%) (all p<0.001). Independent pharmacies had higher prices in neighborhoods with greater proportion of Medicare beneficiaries (+7.5%) and lower prices in neighborhoods with more White residents (-8.7%) (all p<0.001).”

**4. Limitation: While the authors stated that “Pharmacy-level price variation was greater for non-insulin generic therapies than insulins or brand name therapies. Chain pharmacies had both lower prices and lesser variation compared with independent pharmacies”, (Results, Abstract), their implication to practice and policy was unclear given the generic drugs had much lower acquisition costs and cash prices, hence, the cost burden to those customers who do not have health insurance is much smaller. In addition, since the U&C price is not the actual price most consumers are paying, it would be more appropriate to span out the “usual and customary prices” or “cash price” in the title to clarify.**

- We have included new material providing the implications of our findings, adding “While traditionally the focus of drug costs for diabetics has been on insulins or brand name therapies, we find that the price of generic therapies can vary considerably. This is important because even though generic therapies are generally cheaper than other therapies, this significant variation suggests that they are the most sensitive to neighborhood-level variation and that even patients using these therapies could face high financial burden based on where they acquire their generic therapies from. Patients facing financial burden from healthcare costs may consider searching for an accessible pharmacy that might offer them the best rates. Our findings suggest this might be particularly true for chain pharmacies that serve regions with a large proportion of patients lacking health insurance, who are more likely to pay cash prices.”
- We agree with providing greater clarity about the prices studied and have added “cash prices” to the title.

**Reviewer #2: I have also uploaded my comments:**

**1. Authors gave a good rationale for the study- differences in prices could affect those without insurance, other cash paying patients and possibly those with deductibles. I have some comments for clarification and/or consideration.**

**2. Can you give a reference for the definition of U&C, I saw a reference by Joey Mattingly that the law 42C.F.R &447.512(b) requires pharmacies to report the U&C when submitting pharmacy claims for both Rx and OTC but not cash paying sales?**

- We have now added a reference from a excellent primer on drug pricing by Joey Mattingly. However, when reviewing 42 CFR 447.512, we have not been able to find differential reporting standards based on claim vs cash payments.

**3. The authors divided the diabetic medications into 3 groups: insulins, non-insulin generics, and brand name non-insulins. What about brand name drugs that are not GLP1s, SGLTs and DPP4s, such as Glucophage? Or brand name insulins? Were they eliminated from the study?**

- Thank you for this comment. We have now added further details, specifically that “Insulins included both generic insulins such as insulin aspart 70/30 and brand name insulins, which were the vast majority. Non-insulin generics did not include brand name formulations such as Glucophage. A total of 136 drug formulations were included in the analysis.

**4. I think cash PPU is a poor measure to compare these drugs since collectively, they are given at different doses and frequencies (some are once a week, twice a day etc) why don’t you ‘cost per day’? Or by class, such as generic biguanides, brand name biguanides, GLP1s etc? The variability will be significantly different.**

- Due to model choice with log link and drug-level fixed effects, we believe that cash PPU is a valid measure to compare drugs across ZCTA3s. Because our model results examine percent changes, the interpretation of the results won’t change even if we used cost per day as a measure. Our model also includes fixed effects at the drug-level which controls for the absolute level of price across drugs. By using drug-level fixed effects, we can use the model coefficients to estimate the cash PPU of drugs, and then multiply that by different doses and frequencies to estimate cost per day. We include an example in the text with estimates of 30-day supply cash prices for 3 diabetes drugs (one from each of our diabetes drug groups). To address this for readers, we added to the methods “Because our model results examine percent changes, the interpretation of the results won’t change even if we used cost per day as a measure. Our model also includes fixed effects at the drug-level which controls for the absolute level of price across drugs. By using drug-level fixed effects, we can use the model coefficients to estimate the cash PPU of drugs, and then multiply that by different doses and frequencies to estimate cost per day.”
- We have added additional details about our methodology, stating “The cash price PPU data is a long right-skewed distribution. Log-link represented a natural choice for our cash price data, where residuals increase with x, and we expected errors to be proportional with the conditional mean. Our approach was derived using the methods outlined by Manning and Mullahy in their paper on estimating log models.(2) From the log link, we performed the modified park test, a modification of the heteroscedasticity test that is modified to help determine the family of distributions to use for a log link function. The test indicated that the Gamma family should be used.”

**5. Can you define chain pharmacies? Do you mean stand-alone pharmacies like CVS or does it include supermarket chains like Walmart? These 2 chain-types have different pricing strategies and should be treated differently.**

- Our third party data identifies independently owned pharmacies from chain pharmacies but does not further break down chain pharmacy types. We agree that not being able to identify stand-alone pharmacies and supermarket chains is a limitation and would be an area for further research. Therefore, we added this as another limitation in our manuscript.

**6. Can you add the rationale for choosing cutoff for extreme outliers at 10X the median?**

- We chose 10x the median as the cutoff for extreme outliers because this was the cutoff used in a [previously published piece](https://www.acpjournals.org/doi/10.7326/M18-1138?url_ver=Z39.88-2003&rfr_id=ori:rid:crossref.org&rfr_dat=cr_pub%20%200pubmed) on the variation in cash price by retail pharmacy type. We have now clarified the reason for this choice and added the reference stating, “To exclude any extreme outliers, we grouped the data by drug, and determined a cutoff of 10x the median, consistent with a prior published analysis from these data.^6^”

**7. Do you have a reference for how you treated variability across neighborhoods on page 5?**

- On page 5, we explain how we interpreted the model results across ZCTA3s using percent changes. The percent change method is a good way to display the impact of a neighborhood variable on the outcome. We changed our language in that paragraph for clarity.

**8. Typo: Tablet instead of table (page 5 last line)**

- Fixed, thank you!

**9. Please use generic names instead of brand names like Lantus and Jardiance.**

- Fixed, thank you!

**10. Because of how broadly you classified the drugs into 3 groups- it weakens the relevance of the findings. It will be better to treat the drugs by class and that will be meaningful for prescribers.**

- Thank you for this comment. This analysis addresses price variability based on type of drug rather than prescriber patterns. While we chose the groups broadly, the model includes drug-level fixed effects. Furthermore, given the fact that price variability was minimal within our groups other than generic non-insulin therapies, it does justify the current classification. However, we provided examples of individual drug price variation in the results to provide context for prescribers.

**6. PLOS authors have the option to publish the peer review history of their article (**[**what does this mean?**](https://secure-web.cisco.com/1ARvd8PdYirE2CtaW4aZ3ToACx-FjlS5118beo1Yex9VIacVSZUY9He1Sy7S165QI030t0IYmhBrZYk_S-_qEwsHUNO_NfmkoTsiOQPFfGE-3MX-6olEZ4mHXI7NNhNmRr_sefHnfeZgu25ielQxe_umSHjQDe5ekcoLrTSMUCu-ShH13V17G3jDVJk450KI6LK0F4lo3b57DILSeKtQ5beDFEdebMdsYBsXngAkCURFZhITm2fa7I5siaC9fQ0bra-PhwE-YRP7p2PgcL4lx4BLp8OyueVbtpZess5uQzYbg94HVi6ck25jr7MeH6v9s/https%3A%2F%2Fjournals.plos.org%2Fplosone%2Fs%2Feditorial-and-peer-review-process%23loc-peer-review-history)**). If published, this will include your full peer review and any attached files.


If you choose “no”, your identity will remain anonymous but your review may still be made public.


Do you want your identity to be public for this peer review? For information about this choice, including consent withdrawal, please see our** [**Privacy Policy**](https://secure-web.cisco.com/19Ao8k2_Aqc_029RVl4kJe7b3m3_aCRn2SBq7g2qQk72eVjJ2djYZyC8dmlcwpPPsTVFnKRAVXVHQ76DZqS0uRiJo4YKoKfPIFJhuEVsUpNYuM0ujWD938AEpcMrj2POZ6kIWKq0ubzYsWlojvJnjfAFhRai_PWlQa5vkFfg_Thm4vZairsveruBqHCSgiDL8uv-IxMrbiTO61OKWtJems_EjidkmkZEYET51WZsQR1gPo8XLgd2Gh2TnE2VEaqnNKaHhsQ0sqZeVxT6Gq9Qd7VmOtiNfpIWA1C14cujTcNnje6Ts7A62WJRcZGGij0Cz/https%3A%2F%2Fwww.plos.org%2Fprivacy-policy)**.**

**Reviewer #1: No**

**Reviewer #2: No**
